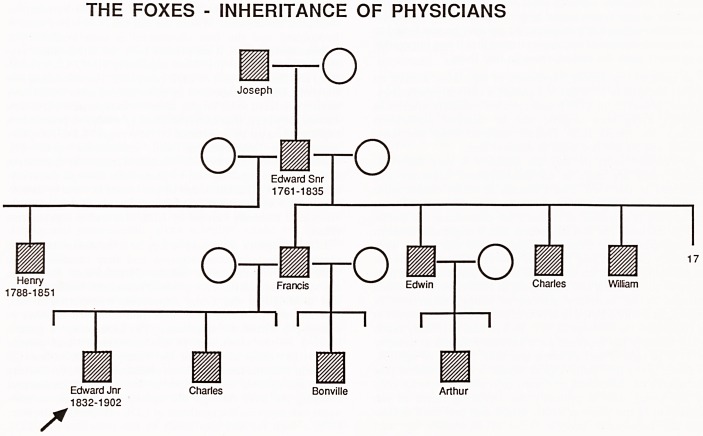# Coronaries, Cholesterol and Children

**Published:** 1990-03

**Authors:** June K Lloyd

**Affiliations:** Nuffield Professor of Child Health, Institute of Child Health, Honorary Consultant Physician, Hospital for Sick Children, Great Ormond Street, London WC1N 3JH


### Coronaries, Cholesterol and Children

### The 1989 Long Fox Lecture

**June K Lloyd MD FRCP**
Nuffield Professor of Child Health, Institute of Child Health, Honorary Consultant Physician, Hospital for Sick Children, Great Ormond Street, London WC1N 3JH

The first Long Fox Memorial Lecture was given by Dr John Beddoe in 1904 (1). In his opening remarks he commented that named lectures seemed either to concentrate on extolling the work and virtues of the distinguished person being honoured or gave an up-to-date and forward looking review of an important scientific topic. He favoured a middle course, though in fact in his own lecture entitled 'The ideal Physician' he did not mention Edward Long Fox until very near the end, and then relatively briefly. In the 85 years since that first lecture the majority of speakers have also chosen the middle way and I shall do the same. If my introduction is rather long it is because I have found reading about Edward Long Fox fascinating and have discovered personal points of contact which I hope you will forgive me for mentioning.

My undergraduate education in good Bristol fashion emphasised the importance of a thorough history and as a paediatrician I know the history must start with the family. Thus my biography of Edward Long Fox junior starts with the family tree (figure 1). This is incomplete partly because I have not obtained full details, and partly because I could not fit in all 22 of Edward's uncles and aunts. It is immediately apparent that a dominant condition runs in this family. A large number of males are doctors and a number of these became physicians. Edward's grandfather, Edward Long Fox senior, was elected a physician at the Bristol Royal Infirmary at the age of 25 and his son Henry followed in his father's footsteps at the age of 28. This family tree bears a superficial resemblance to a family tree I shall show later, where familial hypercholesterolaemia has resulted in the early onset of coronary heart disease; in both situations, females would appear to be protected from the genetic influence but the reasons are, of course, very different!

To return to the Foxes; with this family history, what chance did Edward junior have? His early education was in Bath and this is the first experience that he and I share. After further schooling in Shrewsbury he went to Oxford where he obtained 1st Class Honours in Natural Sciences in 1853; exactly 100 years later, and in an entirely different class, I was a paediatric house-physician at the Radcliffe Infirmary. Edward started his medical training in Edinburgh, probably because his grandfather and uncle Henry had graduated there, but (for reasons that I have not been able to find out) he shortly left to come to London in 1854. There he was clinical clerk to Bence Jones at St George's Hospital and he must have sat in the Lecture theatre at Hyde Park Corner where I gave my first lecture to St George's medical students in 1975. Others have pointed out that Edward was a man ahead of his times and it is not surprising that he decided to add paediatrics to his undergraduate experience. This did not at that time figure at all in the St George's curriculum and Edward therefore went to the Hospital for Sick Children at Great Ormond Street where he studied under the founder of

THE FOXES - INHERITANCE OF PHYSICIANS

that great hospital, Dr Charles West. Although I now work at Great Ormond Street I am pleased to say that my patients are not in the same wards where Edward learnt about children's diseases in the middle of the last century.

Edward graduated in medicine in 1857. Postgraduate training was very different in those days; he was immediately appointed physician at the Bristol Royal Infirmary equalling his grandfather's record of achieving this distinction at the age of 25. The conditions of his appointment, however, obliged him to retire after 20 years—"Achieving a Balance" must have been a somewhat easier task in those days! Edward Long Fox was clearly a humble man. We are told that on one of his earliest ward rounds he turned to his students—there were 6 of them in those days—and said "I wish to say that as I have only just passed out of the student stage myself, I shall feel greatly pleased should any of you notice anything overlooked in my walk and practice here that might be of importance in the treatment of cases, if you will kindly remind me of the fact; for by such means we shall be serving the patients as well as helping one another". This must surely represent the very best type of clinical audit.

On this firm foundation Edward built a great career. He was elected a Fellow of the Royal College of Physicians of London, delivered the Bradshaw Lecture in 1882, became a Member of the Royal Medical and Chirurgical and Neurological Society, a President of the Bristol Medico-Chirurgical Society, and in 1894 President of the British Medical Association. Within medicine he chose to specialise in neurology and his books on "The Pathological Anatomy of the Nervous Centres" and "The Influence of the Sympathetic on Disease" were standard works in his times. His own health was complicated by attacks of gout and in his last years by diabetic neuropathy. He died in 1902 at the age of 70 years.

At the time of Edward Long Fox's death, coronary heart disease was considered to be a rare problem; certainly it was seldom recognised or regarded as an important subject for study. The first clear report of coronary occlusion as a cause of sudden death appeared in 1700 (2).

> "A fat poet after a hearty meal and much orating, climbed a flight of stairs, was siezed by great discomfort in his chest and died within a few minutes. At autopsy he was found to have such narrowed coronary arteries that it was impossible to insert even the end of a needle into them."

In spite of this report, Herbeden in his classic lecture on angina pectoris in 1768 did not deduce a coronary pathology. Jenner, however, in 1770 found calcified coronary arteries in patients dying with angina, but he delayed publication because his friend John Hunter suffered from the same malady and he did not want to distress him.

A hundred years on and the situation is very different. Death rates are high in all industrialised countries and especially in our own. Heart disease in males causes more years of working life lost than any other condition. Prevention is a matter of concern for physicians, the public and parliament. The first suggestion that it might also concern paediatricians came from pathologists, and this would have especially pleased Long Fox who always laid great emphasis on pathology and often started his clinical teaching in the post mortem room. Enos and colleagues in 1953 reported marked changes in the coronary arteries of young, apparently fit American soldiers killed in action in Korea (3); Macnamara *et al* confirmed the findings in 1971 in Vietnam (4); Stary much more recently has reported early coronary lesions at autopsy in 17% of infants and children less than 5 years of age (5). Without going into pathological details it can be stated that the uptake of cholesterol into mast cells and endothelial cells of the vessel wall is probably the initiating factor in the formation of the atherosclerotic lesion and that both uptake and the subsequent process occur at an earlier age and advance more rapidly in individuals with raised levels of cholesterol in their plasma. A recent report from the Bogalusa Heart Study in the USA has shown aortic fatty streaks in young people dying before the age of 25 years (mean age at death 18 years) to be strongly related to ante-mortem levels of both total and low density lipoprotein cholesterol (6).

So we come to cholesterol, whose plasma concentrations are determined by both genetic and environmental factors. Any consideration of strategies to control plasma cholesterol and thus influence the development of coronary atheroma demands some understanding of cholesterol metabolism, and I shall therefore give a very brief review.

### CHOLESTEROL METABOLISM

The major transport protein from which cholesterol is taken up by endothelial cells is low-density lipoprotein (LDL), but the other lipoproteins are also all involved in the transport pathways for cholesterol. Lipid metabolism can conveniently be divided into exogenous and endogenous compartments; in the exogenous pathway dietary cholesterol together with dietary triglyceride is incorporated into chylomicrons whose apoproteins comprise apo B 48, apo C and apo E. The triglyceride of the chylomicron core is hydrolysed by lipoprotein lipase at the endothelial surface of capillaries and the remnant, which is relatively cholesterol rich, is taken up by specific receptors on liver cells. Endogenously synthesised cholesterol and triglyceride are secreted from the liver in very-low-density lipoproteins (VLDL) whose core contains relatively large amounts of triglyceride and smaller amounts of cholesterol. The surface apoproteins are apo B 100 (twice the size of the apo B of chylomicrons), apo C and apo E. The triglyceride is hydrolysed in a manner similar to that in chylomicrons and the shrunken particle loses its apoprotein C and is known as intermediate density lipoprotein (IDL). Some of these particles are taken up directly by the liver, but the majority lose most of their remaining triglyceride together with their apoprotein E and become LDL whose core is now composed mainly of cholesterol esters. About two thirds of the LDL is taken up by hepatic and other cells by receptor mediated endocytosis. Within the cell the cholesterol ester is hydrolysed and the free cholesterol is then available for cellular metabolism; it suppresses both the intracellular synthesis of cholesterol (by down-regulating HMG CoA reductase) and the formation of LDL receptors. The remaining one third of LDL is metabolised by other mechanisms which have been considered to be largely receptor independent. It is now known, however, that LDL modified by oxidative processes is rapidly taken up by acetylated or "scavenger" LDL receptors present on macrophages and endothelial cells (7). Modification of LDL by endothelial and mast cells themselves also promotes rapid uptake by these cells through the acetylated receptor. Oxidation can be prevented *in vitro* by antioxidants such as vitamin E, and it is probable that *in vivo* the vitamin E normally carried by LDL also exerts a protective effect.

LDL receptors thus play a key role in determining the plasma cholesterol concentration and they themselves are controlled by both genetic and dietary influences. Receptors are synthesised in the endoplasmic reticulum, transported to and modified in the Golgi apparatus, transported to and inserted into the membrane, and then collected together in coated pits within the membrane. The LDL receptor gene is located on the short arm of chromosome 19 and genetic defects can occur at each of the 4 major steps; at least 29 different mutants have now been characterised (8, 9). Dietary influences operate in a number of ways: dietary cholesterol entering the liver through the uptake of chylomicron remnants can suppress the synthesis of LDL receptors in the liver (10); a high fat diet can result in the secretion of VLDL

particles as well as chylomicrons from the intestine (11) and thus increase the pool of circulating IDL and LDL; and saturated fatty acids can directly suppress LDL receptor activity (12). Conversely a high intake of polyunsaturated fatty acids (notably linoleic acid) may enhance the activity of LDL receptors thereby decreasing plasma concentrations. The main effect of dietary fatty acids, however, is probably not mediated through receptor clearance of LDL but by increasing (for saturated fatty acids) or decreasing (polyunsaturated fatty acids) the synthetic rates of apo B (14).

### DIET AND PLASMA CHOLESTEROL IN CHILDREN

In children most of the detailed studies of the influence of dietary fat and cholesterol upon plasma cholesterol concentrations have been made in infants fed on human milk and various commercial formulas. Concentrations of all plasma lipids and lipoproteins are low at birth, and in healthy milk fed babies total cholesterol levels rise rapidly during the first week of life and continue to rise until about 4 months when levels plateau out (15). No correlation has been found between plasma cholesterol concentrations at birth and at one year (15, 16). This is true also for low-density lipoprotein (LDL) cholesterol, high-density lipoprotein (HDL) cholesterol, and for triglyceride. Plasma cholesterol and LDL concentrations at 6 months of age do, nevertheless, correlate with levels at one year, the correlation coefficient in the Bogalusa study being about 0.42 (16). The total fat intake of babies is fairly constant whether they are fed human (breast) milk or an industrially produced infant formula both of which contain about 35 g/l. The composition of the fat will, however, vary greatly especially with respect to polyunsaturated fatty acids such as linoleic acid. In human milk levels of linoleic acid are dependent upon maternal intake and can range from around 8% of total fatty acids to as much as 25%; the modified infant formulas currently used contain about 15–20%. The cholesterol intake of infants also varies considerably according to the method of feeding. Human milk contains around 200–300 g/l; unmodified cow's milk has about 70–140 g/l and modified formulas even less at below 50 g/l (17) because of the replacement of much of the butter fat by vegetable oils.

During the period of predominately milk feeding, that is the first 4 to 6 months of life in most industrialised societies, there is a clear correlation between plasma cholesterol concentrations and the type of feed. Babies fed human milk have higher levels than those fed on formula with a stronger correlation between a low polyunsaturated/saturated fat ratio than with a high cholesterol content (18, 19). Once mixed feeding is established the difference disappears and no effect of early feeding on plasma cholesterol concentrations can be detected in later infancy and childhood (18, 19). A study designed to investigate the effect of dietary cholesterol intake during the first year of life also showed no differences at one year of age between infants initially fed on a low cholesterol intake and subsequently given a higher intake on the one hand, and those fed on a higher intake throughout on the other (20).

Studies on the longer term effect of early infant feeding on plasma cholesterol are difficult to interpret. In a study of 97 American school children age 7–12 years, higher mean concentrations were found in those fed on human milk during the first 3 months of life than in those fed on low cholesterol (formula) feed, although the current diet was not different (21). By contrast, analysis of the plasma cholesterol of 172 subjects in the UK aged 32 years of age showed that the women who had been breast fed had significantly lower levels than women who were formula fed; for men the difference was not significant (22).

In spite of the lack of direct evidence in man linking nutrition and plasma lipids in the first year of life with the later development of atherosclerotic heart disease, experimental observations in non-human primates support the concept that "programming" of lipid metabolism may occur in early infancy. Studies in baboons have shown that animals fed on maternal milk (about 300 mg/l cholesterol) for the first 4 months of life had higher plasma LDL and more atherosclerotic lesions in adult life than animals fed on formulas with differing cholesterol concentrations (23). Such experiments clearly indicate that "programming" in early infancy can occur and also remind us that breast feeding, so important for health in early life, has not necessarily evolved to confer longevity or good health in the post reproductive period (24).

The role of diet in causing and maintaining raised levels of plasma total cholesterol and LDL in older children is now generally accepted, with a high intake of total fat and cholesterol, and a low polyunsaturated to saturated fatty acid ratio, being the major factors. The evidence is, however, based on epidemiological studies of populations; within such populations the correlations between nutrient intake and the concentrations of plasma lipids and lipoproteins, though statistically significant, tend to be rather small (25). This is probably due, at least in part, to the inherent problems of dietary recall studies. Nevertheless, the difference between diet and plasma cholesterol concentration in various countries is so great that the role of diet cannot be neglected. In a study of 560 boys aged 7 to 8 years in 16 countries from different regions of the world selected on the basis of having different patterns of diet and different rates of mortality from coronary heart disease, Knuiman *et al* (26) found a strongly positive correlation between the levels of total cholesterol in the children and the prevalence of coronary heart disease in the adults. There was also a high correlation between mean plasma cholesterol concentration and the availability of animal products, and by inference a high saturated fat intake.

### GENES AND PLASMA CHOLESTEROL

Genetic control of plasma cholesterol is mediated through genes responsible for the synthesis of the various apoproteins and the LDL receptor. The commonest cause of raised cholesterol is due to the interaction of environmental factors, of which diet (as already discussed) is probably the most important, and a number of genes—so called polygenic inheritance. Of the monogenic disorders, that affecting the LDL receptor and resulting in the disorder known as familial hypercholesterolaemia is the condition most likely to be expressed during the childhood years (9). The gene frequency in the Caucasian population is of the order of 1 in 500 which makes it one of the most common dominantly inherited conditions. Heterozygous individuals can be diagnosed in infancy and have raised levels of total cholesterol and LDL cholesterol but usually no clinical abnormality. The detection of children therefore depends upon testing as a result of recognising the significance of the onset of coronary heart disease at an early age in a family member. The risks for coronary heart disease in heterozygotes are greater and occur at an earlier age in males than females with about 50% of the men experiencing their first episode of ischaemic heart disease by the age of 50 years (27). Although familial hypercholesterolaemia accounts for only a small proportion of total coronary heart disease it is particularly important in younger people, and because it is expressed fully in childhood its detection and management at this age assumes considerable importance. The homozygote form of familial hypercholesterolaemia is very rare and extremely serious Tendinous and tuberous xanthoma and corneal arcus are found in early childhood and clinical evidence of coronary heart disease is often evident early in the second decade (28). Untreated individuals seldom survive beyond the age of 30 years.

The other dominantly inherited disorders of lipoprotein metabolism—the so-called mixed hyperlipidaemias—cause both hypercholesterolaemia and hypertriglyceridaemia and are certainly associated with coronary and peripheral vascular disease in adult life. They are, however, only rarely expressed biochemically during the childhood years (29) and thus preventive measures are difficult to apply to specific children.

### CONTROL OF HYPERCHOLESTEROLAEMIA IN CHILDHOOD

Because the justification for detecting and attempting to treat hypercholesterolaemia in children rests largely on its identification as a risk factor for coronary heart disease in adults it is pertinent to consider some rather basic questions before embarking on any strategy for the childhood population. First and most importantly, if we identify a high plasma cholesterol level in a child will this persist into adult life. In familial hypercholesterolaemia the answer is in the affirmative, although even here the diagnosis may be difficult to establish during the first year of life or in cases where cholesterol levels are "borderline". For children whose hypercholesterolaemia results from polygenic and environmental casuses the certainty that levels will remain within the same centile ranking (tracking) is less secure. The probability increases with increasing age (Boulton, personal communication) and by the end of the first decade is of the order of 0.54–0.75 (30).

The second question relates to the certainty with which we can claim that lowering of plasma cholesterol will prevent or delay the onset of coronary heart disease. For the childhood population there is at present no evidence but primary prevention studies in adults strongly suggest benefit (31), and results of treating the rare homozygous form of familial hypercholesterolaemia support this (32, 33).

The third question relates to treatment itself; plasma cholesterol concentrations can certainly be reduced in children by dietary modification and if necessary by drugs. Maintenance of such regimes on a long-term, indeed life-long, basis is however less certain. Studies in familial hypercholesterolaemia indicate that the majority of affected children fail to comply with dietary treatment after about 2 years and only 30% remain on the currently most effective drug (cholestyramine) by 8 years (34).

This leads to the final question—should the approach to the control of hypercholesterolaemia be selective and if so how should selection be achieved. For the childhood population in general there would appear to be no justification for universal screening by estimation of plasma cholesterol; such measurements are not sensitive enough to diagnose familia hypercholesterolaemia and tracking is not sufficiently strong for risk to be clearly identified in childhood. Nevertheless, some modification of the current high fat diet of so-called developed populations can be introduced during childhood. There is general agreement that no change in current practice is indicated in the first 2 years of life but thereafter recommendations have been made by official bodies in a number of countries. An expert committee of the Department of Health in the United Kingdom has recommended that 35% of food energy should be derived from fat with only 15% coming from saturated fatty acids but this change was not intended to apply to children under 5 years who should continue to receive whole cows' milk (35). This committee made no specific recommendations on cholesterol intake although other bodies have suggested a limit of 250–300 mg/day (36). Further recommendations included compensating for the reduced fat intake with increased fibre-rich carbohydrates and avoiding any future increase in sucrose or common salt. The suggestion that only whole milk should be given to children under 5 years is at variance with a number of other reports; in both Sweden (37) and Canada (38) the use of semi-skimmed milk (fat content about 2%) has not had any deleterious effects, and further guidelines in the UK have stated that semi-skimmed milk may be introduced into the diet of children between the ages of 2 to 5 years provided that the diet as a whole is nutritionally adequate (39).

In industrialised countries where fat intake, and particularly fat high in saturated fatty acids, has risen sharply over the past decades, the current trend towards a decreased consumption of saturated fats, cholesterol and salt and an increased intake of polyunsaturated fats can probably be followed in moderation by older children though extremes should be avoided (40). Such diets, however, must be nutritionally adequate and should provide the basis of a "healthy eating lifestyle". The Committee on Nutrition of the American Academy of Pediatrics emphasise that "any recommendation for changing towards a more restictive dietary pattern during the first two decades of life should await demonstration that such dietary restrictions are needed and, in addition, that such restrictions would support adequate growth and development for children and adolescents" (40).

For children at special risk, that is those with familial hypercholesterolaemia, the situation is different. Selective screening targetted on the family should be undertaken (41). Nevertheless, treatment of these chldren remains difficult. Dietary management alone is often inadequate and cholestyramine, the current drug of choice, is unpalatable. The advent of the new generation of HMV CoA reductase inhibitors, however, raises real hope for these individuals;' the drug is already licenced for use in adults and trials in children may start in 1–2 years.

In this lecture I have chosen to emphasise the role of cholesterol in the causation of coronary disease. It would be inappropriate to end, however, without remembering that it is only one of the risk factors identified in adults to which attention should be paid in childhood. Kannel & Dawber (42), nearly 20 years ago, proposed 5 items to which paediatricians should pay attention—hyperlipidaemia, hypertension, obesity, cigarette smoking, and physical inactivity. I would like to go even further back to the 1926 Long Fox Lecture given by Dr Carey Coombs (43). His subject was the aetiology of cardiac disease and his conclusions are as true today as they were half a century ago: he shall have the last word.

> ". . . diseases of the heart arise, not from single causes only, but from conspiracies of causes. It is not the seed alone that matters but also the soil and the weather. . . . the hope of advance lies in prevention rather than in cure."

### REFERENCES

1. BEDDOE, J. (1904) The Long Fox Lecture. *Bris. Med-Chir. J.* **2**, 303–320.
2. BONETUS quoted in WHITE, P. (1955) Coronaries through the ages. *Minnesota Medicine* **38**, 801–808.
3. ENOS, W. F., HOLMES, R. H., BEYER, J. C. (1953) Coronary disease among United States soldiers killed in action in Korea. *JAMA* **152**, 1090–1093.
4. MACNAMARA, J. J., MOLOT, M. A., STREMPLE, J. F., CUTTING, R. T. (1971) Coronary artery disease in combat casualties in Vietnam. *JAMA,* **216**, 1185–1189.
5. STARY, H. E. (1987) Macrophages, marcrophage foam cells and eccentric internal thickening in the coronary arteries of young children. *Atherosclerosis* **64**, 91–108.
6. NEWMAN, W. P., III, FREEDMAN, D. S., VOORS, A. W. *et al.* (1986) Relation of serum lipoprotein levels and systolic blood pressure to early atherosclerosis: The Bogalusa Heart Study. *New Engl. J. Med.* **314**, 138–144.
7. STEINBERG, D., PARTHASARTHY, S., CAREW, T. E. *et al.* (1989) Beyond cholesterol: Modifications of low-density lipoprotein that increase its atherogenicity. *New Engl. J. Med.* **320**, 915–924.
8. HOBBS, H. H., LEITERSDORF, E., LEFFERT, C. C. *et al.* (1989) Evidence for a dominant gene that suppresses hypercholesteraemia in a family with defective low-density lipoprotein receptors. *J. Clin. Invest.* **84**, 656–664.

9. GOLDSTEIN, J. L., BROWN, M. S. (1989) Familial hypercholesterolaemia. *In*: The Metabolic Basis of Inherited Disease. Ed. C. R. SCRIVER, A. L. BEAUDET, W. S. SLY and D. VALLE. 6th edn. New York: McGraw-Hill Book Co. 1215–1250.
10. GOLDSTEIN, J. L., BROWN, M. S. (1982) Lipoprotein receptors; genetic defense against atherosclerosis. *Clin. Res.* **30**, 417–426.
11. EDELIN, Y. H., KINSELL, L. W., MICHAELS, G. D., SPLITTER, S. D. (1968) Relationship between dietary fat and fatty acid composition of "endogenous" and "exogenous" very-low-density lipoprotein triglycerides. *Metabolism* **17**, 544–550.
12. SPADY, D. K., DIETSCHY, J. (1985) Dietary saturated triglyceride suppresses hepatic low density lipoprotein receptors in the hamster. *Proc. Natl. Acad. Sci. USA* **82**, 4526–4530.
13. SHEPHERD, J., PACKARD, C. J., GRUNDY, S. M. *et al.* (1980) Effect of saturated and polyunsaturated fat diets on the chemical composition and metabolism of low density lipoproteins in man. *J. Lipid Res.* **21**, 91–99.
14. CORTESE, C., LEVY, J., JANUS, E. D. *et al.* (1983) Modes of action of lipid-lowering diets in man: Studies of apolipoprotein B kinetics in relation to fat consumption and dietary fat composition. Europ. J. Clin. Invest. *13*, 79–85.
15. DARMADY, J. M., FOSBROOKE, A. S., LLOYD, J. K. (1972) Prospective study of serum cholesterol levels during the first year of life. *Brit. Med. J.* **2**, 685–687.
16. BERENSON, G. S. (1980) Cardiovascular Risk Factors in Children. Oxford: Oxford University Press. Ch. 10, pp. 166–198.
17. Guidelines on Infant Nutrition I. *Acta. Paed. Scand. Suppl.* (1977) 262, p. 10.
18. ANDERSEN, G. E., LIFSCHITZ, C., FRIIS-HANSEN, B. (1979) Dietary habits and serum lipids during the first 4 years of life. *Acta. Paed. Scand.* **68**, 165–170.
19. HUTTUNEN, J. K., SAATINEN, U. M., KOSTIAINEN, E., SIIMES, M. A. (1983) Fat composition of infant diet does not influence subsequent serum lipid levels in man. *Atherosclerosis* **46**, 87–94.
20. GLUECK, C. J., TSANG, R., BALISTERI, W., FALLAT, R. (1977) Plasma and dietary cholesterol in infancy: Effects of early or moderate cholesterol intake on subsequent response to increased dietary cholesterol. *Metabolism* **21**, 1181–1192.
21. HODGSON, P. A., ELLEFSON, R. D., ELVEBACK, L. R., HARRIS, L. E., NELSON, R. A., WEIDMAN, W. H. (1970) Comparison of serum cholesterol in children fed high, moderate, or low cholesterol milk diets during the neonatal period. *Metabolism* **25**, 739–746.
22. MARMOT, M. G., PAGE, C. M., ATKINS, E., DOUGLAS, J. W. B. (1980) Effect of breast-feeding on plasma cholesterol and weight in young adults. *J. Epidemiol. Comm. Health* **34**, 164–167.
23. MOTT, G. E. (1986) Deferred effects of breast feeding versus formula feeding on serum lipoprotein concentrations and cholesterol metabolsm in baboons. *In*: FILER, L. J. Jr, FOMON, S. J. (eds). The Breastfed Infant: a model for performance. Ninety-first Ross Conference on Pediatric Research. Columbus, Ohio. Ross Laboratories 144–149.
24. LUCAS, A. (1978) Diet in early life; evidence for its later effects. *In*: Infant Nutrition and Cardiovascular disease. MRC Environmental Epidemiology Unit. Scientific Report No. 8. Southampton, 11–16.
25. FRANK, G. C., BERENSON, G. S., WEBBER, L. S. (1978) Dietary studies and the relationship of diet to cardiovascular risk factor variables in 10 year old children; the Bogalusa Heart Study. *Amer. J. Clin. Nutr.* **131**, 328–340.
26. KNUIMAN, J. T., HERMUS, R. J. J., HAUTVAST, J. G. A. J. (1980) Serum total and high density lipoprotein (HDL) concentration in rural and urban boys from 16 countries. *Atherosclerosis* **36**, 529–537.
27. SLACK, J. (1969) Risks of ischaemic heart disease in familial hyperlipoproteinaemic states. *Lancet* ii, 1380–1382.
28. SPRECHER, D. S., SCHAEFER, E. J., KENT, K. M. *et al.* (1984) Cardiovascular features of homozygous familial hypercholesterolaemia. Analysis of 16 patients. *Am. J. Cardiol.* **54**, 20–32.
29. GLUECK, C. J., FALLAT, R., BUNCHER, C. R. *et al.* (1973) Familial combined hyperlipoproteinaemia: Studies in 91 adults and 95 children from 33 kindreds. *Metabolism* **22**, 1403–1428.
30. KWITEROVICH, P. O. Jr (1986) Biochemical, clinical, epidemiologic, genetic and pathologic data in pediatric age group relevant to the cholesterol hypothesis. *Pediatrics* **78**, 349–362.
31. Lipid Research Clinics Program: Primary prevention trials. *JAMA* (1984) **251**, 351–374.
32. STARZL, T. E., CHASE, H. P., AHRENS, E. H. Jr *et al* (1983) Portocaval shunt in patients with familial hypercholesterolaemia. *Ann. Surg.* **198**, 273–283.
33. WEST, R. J., GIBSON, P. E., LLOYD, J. K. (1985) Treatment of homozygous familial hypercholesterolaemia: An informative sibship. *BMJ* **291**, 1079–1080.
34. WEST, R. J., LLOYD, J. K., LEONARD, J. V. (1980) Long-term follow-up of children with familial hypercholesterolaemia treated with cholestyramine. *Lancet* **2**, 873–875.
35. Diet and Cardiovascular Disease. Committee on Medical Aspects of Food Policy. Report on Health and Social subjects No. 28. (1984) London: H.M. Stationery Office.
36. WEIDMAN, W., KWITEROVICH, P. Jr., JESSE, M. J. *et al.* (1983) Diet in the healthy child: AHA Nutrition Committee Report. *Circulation* **67**, 1411A–1414A.
37. HAGMAN, U., BRUCE, A., PERSSON, L., SAMUELSON, G., SJOLIN, S. (1986) Food habits and nutrient intake in childhood in relation to health and socio-economic conditions: a Swedish multicentre study, 1980–81. *Acta. Paed. Scand. (Suppl.)* **328**, 4–56.
38. LEUNG, D. L., PENNELL, M. D., LEUNG, M., HALL, J. (1982) The effects of a 2% fat milk intake on infant nutrition. *Nutrition Research* **2**, 651–660.
39. Present Day Practice in Infant Feeding: Third Report. Report on Health and Social Subjects, No. 32. (1988) London: H.M. Stationery Office.
40. Committee on Nutrition, American Academy of Pediatrics. Prudent life-style for children: Dietary fat and cholesterol Pediatrics. (1986) **78**, 512–525.
41. LLOYD, J. K., WEST, R. J. (1986) Childhood prevention of coronary heart disease. *Postgrad. Med. J.* **62**, 97–100.
42. KANNEL, W. B., DAWBER, T. R. (1972) Atherosclerosis as a pediatric problem. *J. Pediatrics* **80**, 544.
43. CAREY F. COOMBS (1926) The aetiology of cardiac disease; the Long Fox Memorial Lecture. *Bristol. Med-Chir. J.* **18**, 1–19.

## Figures and Tables

**Figure f1:**